# A service evaluation of the recognition, care and management of functional seizures within a UK ambulance service

**DOI:** 10.29045/14784726.2026.6.11.1.43

**Published:** 2026-06-01

**Authors:** David Williams, Kim Kirby, Elizabeth Mallam, Penny Crawley

**Affiliations:** University of the West of England Bristol ORCID iD: https://orcid.org/0009-0006-1860-8112; University of the West of England Bristol ORCID iD: https://orcid.org/0000-0002-8092-7978; North Bristol NHS Foundation Trust; University of the West of England Bristol

**Keywords:** dissociative seizure, functional neurological disorder, functional seizure

## Abstract

**Introduction:**

Functional seizures, a subtype of functional neurological disorder, are frequent presentations within the emergency department and wider hospital setting. There is a lack of research exploring pre-hospital care for this patient group. The aim of this service evaluation was to explore ambulance staff recognition, care and management of functional seizure presentations within a UK ambulance service trust.

**Methods:**

An online survey was conducted using a convenience sample of clinical staff from South Western Ambulance Services NHS Foundation Trust. Respondents were asked for a mixture of distinct and free-text answers to questions around identification, care and management of functional seizure presentations. Results were analysed using a mix of descriptive statistics and qualitative content analysis.

**Results:**

There were 96 responses to the survey. Eighty-two per cent (n = 81) of respondents reported that they attend functional seizures at least once monthly, with 18% (n = 17) of those attending functional seizures weekly. Ambulance staff confidence in the recognition and management of functional seizures is high, but notably 66% (n = 63) have received no previous training or education for functional seizure care. Definitions and causes of functional seizures reportedly relied predominantly on a psychological component. Identification was based on history, seizure features and clinical guidelines. Management of functional seizures focused on reassurance. Challenges identified by ambulance staff included stigma, perceived diagnostic complexity, fear and limited education.

**Conclusion:**

Ambulance staff frequently attend functional seizures but receive limited training, leading to misconceptions, stigma and diagnostic challenges. Identification depends on unreliable signs and complex histories. Management aligns with clinical guidance, yet uncertainty remains for prolonged events. Fear of misdiagnosis, harmful attitudes and poor education highlight the urgent need for targeted education and future research.

## Introduction

Functional neurological disorders (FNDs) are defined by the National Institute for Health and Care Excellence (NICE) as ‘a condition in which people experience neurological symptoms in the absence of any identifiable causative physical or structural abnormality’ (NICE, 2019). FND is a highly prevalent condition, estimated to affect 50,000–100,000 people in the UK (Finkelstein et al., 2025), and it accounts for approximately 9% of acute neurological hospital admissions (Beharry et al., 2021), highlighting its significance as an acute clinical presentation.

Despite the lack of a structural brain abnormality in FND, symptoms are genuine and involuntary (Edwards et al., 2023). FND may present with a range of symptoms, including motor, sensory and cognitive dysfunction (Hallett et al., 2022). Functional seizures are a common subtype that frequently present to the emergency department (ED) (Finkelstein et al., 2021). However, patient guidance suggests that for individuals with a diagnosis of functional seizures, an ambulance is not usually required and that many episodes can be managed safely in the community (Epilepsy Action, 2024).While studies have explored care of functional seizure patients within the ED setting, there is an absence of research focused on care provided to this patient group by pre-hospital clinicians (Finkelstein et al., 2021; Higson et al., 2025; Lehn et al., 2021).

This service evaluation aimed to explore South Western Ambulance Service NHS Foundation Trust (SWASFT) ambulance clinicians’ knowledge of and exposure to functional seizures, their confidence, their attitudes towards patients with functional seizures and the challenges they encountered in the recognition and management of functional seizures in the pre-hospital setting.

## Methods

### Study design and data collection

This was a service evaluation undertaken using an online survey on the Qualtrics platform (Qualtrics, 2024). The original order and wording of the questions given to respondents in the survey can be found in Supplementary 1. Eligible respondents were defined as clinical staff, either in a frontline or emergency operations centre (EOC) role, working within SWASFT. A convenience sample was obtained by advertising the survey via the internal staff bulletin and SWASTCPD website.

The survey consisted of a combination of Likert scale questions, open-ended free-text responses and fixed-response categorical items. Survey results were collected over a six-week period (20 February to 3 April 2025).

### Data analysis

Distinct questions, such as role or length of service, were analysed using a descriptive approach, whereas for free-text answers, qualitative content analysis was utilised to interpret the data. Data were coded and themes generated by a single author using NVivo qualitative data analysis software (Lumivero, 2023).

### Governance

The Health Research Authority (HRA) decision tool did not deem the study to be research; therefore HRA approval was not sought (HRA, 2022). An information sheet was provided to participants within the survey, which explained that responses could not be withdrawn after submission due to anonymisation. Approval was provided by the SWASFT Research and Development Group to undertake this service evaluation. Results were stored securely using a password-protected system.

## Results

A total of 96 responses were collected. Respondents held a range of roles within SWASFT. The majority (76%; n = 73) were in a role requiring health care professional registration. In terms of length of service, 52% (n = 50) of respondents had over 10 years of experience, with just 5% (n = 5) of respondents having two or fewer years of experience.

For frequency of attendance to functional seizures, 82% (n = 81) of respondents identified that they attend patients experiencing functional seizures at least once a month, with 18% (n = 17) of those attending functional seizures weekly.

Respondents were asked to rate their confidence using a Likert scale against four statements (Table 1). Confidence was consistently reported as high, with the majority responding as ‘quite confident’ or ‘extremely confident’ against each statement. The statement with the highest number of respondents reporting either under-confidence or no confidence was discharge on scene.

**Table 1.** Result for ‘Question 7. Please rate your confidence against the following statements’.

Statement	No confidence	Under-confident	Somewhat confident	Quite confident	Extremely confident
Recognising the difference between a functional seizure and an epileptic seizure	1	19	24	39	13
Managing a functional seizure	0	11	13	50	22
Identifying when a patient experiencing a functional seizure can be safely discharged on scene	4	19	19	40	14
Identifying when a patient experiencing a functional seizure needs to be conveyed to the emergency department	1	18	16	49	12

In terms of training and education, 34% (n = 33) of respondents had received previous training or education on functional seizures. Of these, the majority received this in a university programme (n = 18); other answers included ‘continuous education outside of my employed role’ (n = 10), ‘continuous education from my employer’ (n = 9) and ‘initial in-service training course’ (n = 4).

### Defining functional seizures and their cause

Respondents identified the non-epileptic nature of functional seizures. There were suggestions that there was no abnormal brain activity in functional seizures and that there was a psychological origin to these presentations.

*Seizure-like activity caused by a non-organic cause, specifically psychological origins. A physical manifestation of underlying psychological distress, stress or trauma, not unlike how an anxiety attack could cause chest pain or breathlessness.* (Respondent 89)

In addition, there were numerous terms used synonymously for functional seizures within the responses, including PNES (psychogenic non-epileptic seizures), conversion disorder and dissociative seizures.

*A psychogenic or dissociative seizure, psychological in origin, subconscious response to emotional trauma.* (Respondent 63)

Several respondents used analogies to try to explain what a functional seizure is, and a minority of respondents suggested functional seizures were either related to behaviour or were attention seeking in nature.

*A seizure of non-epileptic cause. It was described to me as ‘programming’ as opposed to ‘wiring’, which is a helpful way of thinking about it. They can come with or without warning and patients do not have the ability to prevent them.* (Respondent 9)

*Non epileptic. Behavioural. Usually affects people with mental health problems who want attention.* (Respondent 70)

However, most respondents suggested functional seizures were involuntary and not the patient’s choice. Several respondents identified that they had poor existing knowledge of functional seizures.

*Who knows. Very complicated question. Do[es] anyone actually know.* (Respondent 61)

### Identifying functional seizures

A prominent topic for identification of functional seizures was the patient’s history and background information on scene. Respondents reported using a previous diagnosis, including a lack of an epilepsy diagnosis, history of an emotional or stress-related trigger, medication prescribed to the patient and access to notes such as GP records to help identify functional seizures.

*History of functional seizures, history of triggers such as overwhelming stress/emotions prior to seizure…* (Respondent 2)

Another trend identified was specific characteristics of the seizure activity, clinical observations and findings during assessment. Characteristics included eyes open or closed, presence of tongue biting or urinary incontinence, presence of an injury, presence of a post-ictal period and whether there was evidence of fluctuating intensity. Clinical observations and assessment findings that supported identification of a functional seizure included oxygen saturation levels, blood glucose, pupillary response, level of consciousness and responsiveness during the episode.

*I often use clinical judgement following a thorough history if the patient is no longer convulsing. Or observe the convulsion myself, I look to see if: eyes open or shut, any tongue biting or incontinence, are the convulsions symmetrical, any rigidity, is there a post-ictal period, any abnormal observations…* (Respondent 18)

Reference to clinician experience and the use of clinical guidelines to support identification was evident. This included frequent mention of the Joint Royal College Ambulance Liaison Committee (JRCALC) seizures guidance.

*JRCALC convulsions in adults guideline has a useful table detailing differences in presentation of a functional and epileptic seizure, whilst I do just know this information now I will always refer to this guideline to double check and confirm to myself that what I am seeing is functional.* (Respondent 38)

*My eyes and over 20 years of seeing seizures.* (Respondent 68)

### Managing functional seizures

Many respondents had a similar approach when a functional seizure episode had been identified if the patient was still experiencing symptoms. This included providing a calm environment, giving reassurance and ensuring that the patient was safe.

*…I offer reassurance to the patient and to anyone they’re with, explain that the seizure they are having is not a type of seizure that is responsive to medication and that our priorities are keeping them safe and calm until the seizure stops…* (Respondent 76)

There were suggestions that once a seizure episode had ceased patients would be discharged on scene if they had a previous diagnosis and this episode was a similar presentation. Conversely, it was suggested that patients experiencing a first episode, or where the diagnosis was unclear, would be conveyed to hospital.

*Often discharge on scene with referral to GP for onward discussion surrounding it. I also point them towards further information online such as the NHS website.* (Respondent 96)

Respondents expressed difficulties in management of patients experiencing functional seizures. For example, uncertainty about what to do if the seizure activity does not stop. Respondents discussing ongoing seizure activity suggested they would convey the patient to the ED if the seizure did not stop while on scene.

*In rare circumstances when patients continue to convulse without recovery despite the above efforts, I have conveyed to an emergency department as unable to manage at scene.* (Respondent 38)

### Challenges faced by ambulance staff

Respondents highlighted that poor attitudes and harmful care exist within the ambulance service for patients who experience functional seizures. Several respondents highlighted problematic terminology for functional seizures, and others provided examples of harmful care. There were also suggestions that poor attitudes extend beyond the ambulance service to the wider healthcare setting.

*There is a lot of stigma around functional seizures, I have heard colleagues say they are ‘faking’ them, I have heard about other paramedics being deliberately cruel to regular patients whilst seizing, using methods such as sternal rubs etc to elicit pain responses, we have a regular who can hear everything whilst experiencing seizures and crew have said/discussed nasty things whilst she was seizing.* (Respondent 33)

*I also actually feel in hospital clinicians have even less understanding around this condition. I have heard of these patients receiving an RSI and ITU care.* (Respondent 38)

Many respondents talked about the complexities of differentiating between epileptic and functional seizures, particularly in episodes that are prolonged and are not ceasing in response to techniques such as reassurance. Similarly, respondents suggested that some patients experience functional seizures with a background diagnosis of epilepsy or other health diagnoses, which further adds to the diagnostic challenge.

*As mentioned before. If no one is with them to tell us their history then you have to treat as presenting. Recognition can often be tricky and over-treatment is common. I don’t always feel confident that I can distinguish between what is and isn’t epileptic as there have been some patients in the past that are very ‘convincing’ in presentation and therefore often hard to know whether to over-treatment or under. No one wants to misdiagnose and not treat an epileptic seizure thinking it’s a PNES.* (Respondent 92)

There was discussion from respondents about fear of getting things wrong.

*Fear of judgement and sanctions should they get the decision/management wrong along with fear of lack of support from the trust should this be the case.* (Respondent 3)

In addition, expectations from family members and members of the public with regards to not providing treatment such as medication in response to the seizure episode were identified.

*Other people that are present such as friends and families may not understand why we aren’t giving medication for functional seizures and expect us to do more.* (Respondent 10)

Finally, respondents frequently identified that a lack of training, knowledge or experience is a significant challenge when recognising or managing functional seizure presentations.

*Inexperienced staff may lack confidence in identifying a functional seizure and therefore may believe that it ‘wouldn’t hurt’ to give pharmacological intervention as they would rather overtreat a functional seizure than undertreat an epileptic seizure.* (Respondent 17)

## Discussion

This service evaluation has identified that ambulance staff exposure to patients experiencing functional seizures is high, with 82% of respondents attending these patients at least once monthly. Clinician confidence in relation to the recognition, management and decision making around conveyance or non-conveyance of functional seizures was reported to be high, but participants reported a lack of training and education. Respondents cited issues around prolonged episodes or complex cases. Additional challenges identified by ambulance staff included stigma, perceived diagnostic complexity, fear and limited education.

### Frequency

Previous data exploring seizure presentations conveyed to the ED by ambulance suggests that approximately 11% of those conveyed to hospital by ambulance are functional seizure presentations (Dickson et al., 2017). However, this does not include patients discharged on scene and managed in the community by ambulance staff. Therefore, given the high reported caseload of functional seizures by ambulance staff in this service evaluation, further work is needed to determine robust prevalence data.

### Definition and cause

In terms of defining functional seizures and their cause, ambulance staff were aware that these events are not epileptic in nature. However, there was a reliance on a history of emotional or psychological trauma in defining functional seizures. Whereas ambulance clinical guidance suggests that a history of post-traumatic stress disorder may help to identify a functional seizure, it is important to note that many patients who experience FND, including functional seizures, will not have a history of emotional or psychological trauma (JRCALC, 2025; Lidstone et al., 2020).

Furthermore, a small number of respondents suggested that functional seizures are a patient’s choice or are behavioural in origin. This is incorrect, and eradicating this perspective is vital for reducing stigma and harm for FND patients (Edwards et al., 2023; Mcloughlin et al., 2024).

While functional seizures are not a patient’s choice, it is possible that there is conflation between FND presentations and patients presenting with malingering or feigning behaviour, as visibly they may appear similar. Expert opinion suggests these presentations are far less common, although the exact prevalence is not clear (Edwards et al., 2023).

### Identification and management

There was discussion about the use of signs, such as non-specific tongue biting or urinary incontinence, to differentiate between a functional and epileptic seizure. However, these signs are not reliable and can occur in both presentations (Brigo et al., 2012, 2013). Similarly, diagnosis of epilepsy to help differentiate between a functional and epileptic seizure should be used with caution, with approximately one in 10 patients experiencing functional seizures with a concurrent diagnosis of epilepsy (Benbadis et al., 2001).

Management of functional seizures generally reflected clinical guidance (Davies et al., 2022; JRCALC, 2025). Clinicians raised questions about what to do if a seizure did not stop and how long to wait on scene with a seizing patient before conveying them to the ED. There is currently no national guidance on what to do in this situation.

There were varying answers about whether to convey the patient to the ED when a functional seizure had ceased. Higson et al. (2025) suggest that ‘development of paramedic functional seizure management guidelines may reduce unnecessary transfer of individuals with functional seizures to ED’. However, national ambulance clinical guidance recommendations regarding conveyance to hospital versus community management are limited, summarising that ‘the decision whether to convey a patient to hospital after a convulsive seizure is difficult’ (JRCALC, 2025).

### Challenges

Respondents identified a range of challenges for ambulance staff. This included poor attitudes and harmful care from other clinicians. Pejorative language was frequently cited and there were also suggestions of harmful care when caring for functional seizure patients. Experience of poor or harmful care is a common occurrence for FND patients (Mcloughlin et al., 2024).

Differentiation between a functional and epileptic event was identified as a key theme within reported challenges. Diagnostic challenges exist in the wider literature and, while some international research has explored rapid identification tools for first responders, further research to evaluate the reliability and practical usefulness of clinical signs presenting in the pre-hospital environment would be valuable (De Paola et al., 2016; Dudley et al., 2024).

Fear around misdiagnosis and the potential harm that incorrect treatment could cause was a significant apprehension for ambulance staff. Such concern among clinicians is consistent with broader findings suggesting that healthcare professionals often experience frustration and uncertainty when managing functional seizures (Rawlings & Reuber, 2018).

Finally, training, knowledge and experience of managing and identifying functional seizures was cited as a challenge. This reflects the wider evidence base, with functional seizure knowledge and education reported as poor across various healthcare professions (Lehn et al., 2020; Milligan et al., 2021).

### Comparison to ED literature

As highlighted at the start of this article, there is a lack of pre-hospital research on functional seizures. However, there is published literature on care for functional seizures in the ED (Cengiz et al., 2024; Finkelstein et al., 2021; Higson et al., 2025; Lehn et al., 2021). The challenges in identification and management of functional seizures that have been identified within this article closely mirror issues highlighted in the existing research. Importantly, this pre-hospital research introduces additional complexities, including length of time to wait on scene during an unresolved functional seizure and the need to manage patient and family expectations in uncontrolled environments.

### Limitations

This service evaluation has several methodological limitations. A convenience sample from a single UK ambulance service was utilised, introducing the risk of sampling and self-selection bias. By the nature of a service evaluation design, the findings were not intended to be generalisable to other ambulance services or wider healthcare contexts. The use of self-reported survey data may also have been influenced by recall bias or misunderstanding of questions, and the mix of Likert-style, fixed-response and open-ended items may not have fully captured participant perspectives. Finally, interpretation of qualitative findings may have been subject to researcher bias.

### Recommendations for future research

Research is needed to generate robust prevalence data and to better characterise the patient cohort presenting to ambulance services with functional seizures. We also recommend exploring clinician attitudes and stigma, evaluating rapid identification and management tools and investigating barriers and facilitators to providing evidence-informed compassionate care.

## Conclusion

This service evaluation has shown that functional seizures are a frequent aspect of ambulance clinicians’ workloads within a UK ambulance service. Although self-reported confidence in recognition and management was high, the findings highlight substantial gaps in education, as well as the presence of misconceptions and stigma that may adversely affect patient care. Nonetheless, clinicians generally adopted approaches consistent with national clinical guidance, focusing on reassurance and patient safety. However, uncertainty remains around the optimal management of prolonged or complex presentations in the pre-hospital environment.

## Additional information

Psychogenic non-epileptic seizure was a common term used by respondents in this service evaluation. However, it should be noted that at the time of the survey data collection this was the term used by national ambulance clinical guidelines, which have recently been updated to refer to these episodes as ‘functional/dissociative seizures’ (JRCALC, 2025).

## Author contributions

DW was responsible for conceptualisation, data collection, analysis, drafting, reviewing and editing. KK was responsible for reviewing, editing and supervision. EM and PC also reviewed and edited the manuscript. DW acts as the guarantor for this article.

## Conflict of interest

None declared.

## Ethics

The HRA decision tool did not deem this service evaluation to be research; therefore HRA approval was not sought. Approval was provided by the SWASFT Research and Development Group to undertake this service evaluation.

## Funding

This service evaluation was supported by a Bristol, North Somerset and South Gloucestershire Integrated Care Board Type 1 Research Capability Funding Grant.

## Statement of generative AI in scientific writing

The authors did not use a generative artificial intelligence (AI) tool or service to assist with preparation or editing of this work.
